# High-Performance Liquid Chromatography Assay for Moxifloxacin in Brain Tissue and Plasma: Validation in a Pharmacokinetic Study in a Murine Model of Cerebral Listeriosis

**DOI:** 10.1155/2012/436349

**Published:** 2012-03-14

**Authors:** Renaud Respaud, Solene Grayo, Eric Singlas, Sophie Dubouch, Alban Le Monnier, Marie-Catherine Lott

**Affiliations:** ^1^Laboratoire de Suivi Thérapeutique et de Contrôle des Médicaments, Service de Pharmacie, Assistance Publique-Hôpitaux de Paris (AP-HP), Hôpital Necker-Enfants Malades, 75015 Paris, France; ^2^Laboratoire de Listeria, Centre National de Référence des Listeria and World Health Organization Collaborating Centre for Foodborne Listeriosis, Institut Pasteur, 75015 Paris, France

## Abstract

Moxifloxacin is a broad-spectrum antibacterial 8-methoxy-fluoroquinolone. In order to evaluate the pharmacokinetic properties of moxifloxacin in mouse plasma and brain tissue, we developed a high-performance liquid chromatography (HPLC) method. This study was based on single-drug delivery, intravenously dosed in a central listeriosis murine model. The method employed a reversed-phase Lichrospher RP-18 with a precolumn (250 × 4.6 mm) and a mobile phase composed of a mixture of acetonitrile, methanol, and citric buffer (pH = 3.5) with sodium dodecyl sulfate and tetrabutylammonium bromide. Fluorescence detection was performed at an excitation wavelength of 290 nm and an emission wavelength of 550 nm. The relative standard deviation of intra- and inter-day assays was <10%. This validated method led to a short retention time (8.0 min) for moxifloxacin. The standard curves were linear from 5–250 **μ**g/L in plasma and from 0.1–2.5 **μ**g/g of brain tissue. The limits of quantification were 5 **μ**g/L in plasma and 0.1 **μ**g/g in brain tissue. The method enabled the detection of systemic antimicrobial in plasma and in CNS in *Listeria*-infected mice. Injected moxifloxacin passed through the encephalic barrier within a 30 to 60 min after injection time frame. Moxifloxacin pharmacokinetics are modeled in an infected model compared to control mice.

## 1. Introduction

Moxifloxacin (1-cyclopropyl-6-fluoro-1,4-dihydro-8-methoxy-7-[(4aS.7aS)-octahydro-6H-pyrrolo-[3,4-b]pyridin-6-yl]-4-oxo-3-quinolinecarboxylic acid hydrochloride, BAY 12-8039) is a fluoroquinolone molecule with a broad antibacterial spectrum of activity encompassing gram-negative and gram-positive bacteria [1, 2].

*Listeria monocytogenes *is a gram-positive bacterium that is widespread in the environment [3]. It is a facultative intracellular food-borne pathogen that causes severe and life-threatening infections, especially septicemia, abortions, and central nervous system (CNS) infections [3]. Listeriosis mainly occurs in high-risk groups, including individuals with severe underlying diseases or with impaired immunity [4]. Importantly, moxifloxacin has rapid bactericidal activity against extracellular and intracellular *L. monocytogenes *[5]; consequently, this drug can be considered more effective than amoxicillin, the reference treatment, which is only bacteriostatic against *L. monocytogenes *[6]. Moreover, Grayo et al. determined minimal inhibitory concentrations (MICs) for a large collection of *L. monocytogenes* strains and did not detect any that were resistant, regardless of their origin [5]. Secondly, they confirmed that moxifloxacin has rapid *in vivo* activity against *L. monocytogenes* in BALB/c mice [7]. To assess the efficiency of moxifloxacin in this murine model of central listeriosis [7], a pharmacokinetic study of this fluoroquinolone in plasma and brain was necessary. Indeed, some results can even be misleading: fosfomycin has recently been declared effective against *L. monocytogenes in vivo*, whereas it is not *in vitro* [8].

In plasma, moxifloxacin quantification methods have been performed to clarify its clinical efficacy [9–12]. Due to the intracellular development of *L. monocytogenes*, it seems more relevant to measure moxifloxacin concentrations in CNS tissue rather than in cerebrospinal fluid (CSF) [13, 14]. However, no bioanalytical assay has been performed to determine moxifloxacin concentrations in brain tissue (CNS). Thus, we developed a high-performance liquid chromatographic (HPLC) method for the determination of moxifloxacin in plasma and brain. Our method was then utilised to evaluate the pharmacokinetic parameters of moxifloxacin in plasma and brain in a validated murine model of CNS listeriosis.

## 2. Experimental

### 2.1. Chemicals and Reagents

**Figure 1:**
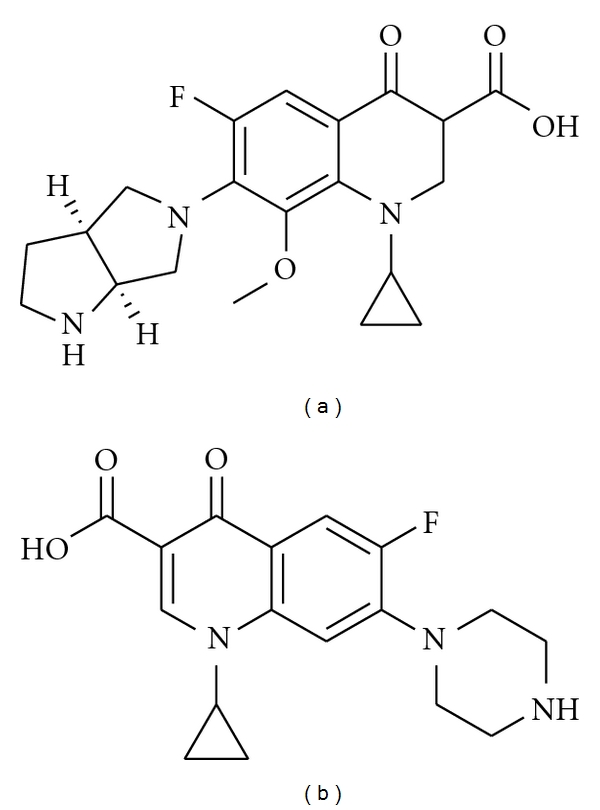
Chemical structures of the fluoroquinolones: (a) moxifloxacin (1-cyclopropyl-6-fluoro-1,4-dihydro-8-methoxy-7-[(4aS.7aS)-octahydro-6H-pyrrolo-[3,4-b]pyridin-6-yl]-4-oxo-3-quinolinecarboxylic acid hydrochloride, BAY 12-8039); (b) ciprofloxacin (internal standard).

Moxifloxacin (Figure 1(a)) and ciprofloxacin (Figure 1(b)) were purchased from Bayer Pharma (Germany and France). Prolabo (Fontenay-sous-Bois/France) provided analytical-grade (Normapur) dipotassium hydrogenphosphate, potassium dihydrogenphosphate and orthophosphoric acid (85%). Sigma (Saint Quentin Fallavier, France) provided the tetrabutylammonium bromide (TBABr). Graded acetonitrile and methanol were purchased from Prolabo.

Stock solutions of moxifloxacin HCl and ciprofloxacin were in methanol (1 g/L): working standard solutions and working internal solutions were in methanol (3 mg/L).

Citric buffer consisted of 25 mM citric acid, 10 mM sodium dodecyl sulfate (SDS), and 10 mM TBABr in 500 mL water; the pH was adjusted to 3.5 with 0.1 M NaOH.

### 2.2. Instrumentation

Chromatographic analysis was performed on a HPLC system consisting of a pump (LC 10A, Shimadzu, Touzart et Matignon, France), thermostat (maintained at 25°C) 717 Plus autosampler Waters, and a RF 551 fluorescence detector (Shimadzu, Touzart et Matignon, France). Separation was executed by a reversed-phase Lichrospher C18 precolumn and column (250 × 4 mm, 5 *μ*m) (Merck, Darmstadt, Germany). All data were analyzed using Chromeleon (version 6.7). The mobile phase consisted of an acetonitrile-methanol-buffer mixture (pH 3.5) (40 : 3 : 57, v/v/v) at a flow rate of 1.0 mL/min. Fluorescence detection was performed at an excitation wavelength of 290 nm and an emission wavelength of 550 nm.

### 2.3. Sample Preparation

To 100 *μ*L of plasma or brain (0.5 g crushed in 0.5 mL of water), 20 *μ*L of the working internal standard solution was added. The samples were deproteinized with acetonitrile (100 *μ*L). After centrifugation (3000 rpm for 10 minutes), the supernatant (100 *μ*L) was mixed with sterile water (400 *μ*L); finally, 20 *μ*L of this solution was injected into the chromatographic system.

For the brain, as described above, we added the ciprofloxacin solution (20 *μ*L) to 100 *μ*L of crushed brain. Then, repeating the plasma sample process, 20 *μ*L of the solution was injected into the chromatographic system.

### 2.4. Method Validation

Validation experiments were designed according to the “Guidance for Industry-bioanalytical Method Validation,” recommended by the US Food and Drug Administration (FDA) [15, 16]. The specificity of the method was evaluated by assaying mouse blank plasma (*n* = 48) and mouse brain tissue (*n* = 6) from separate animals. As validation of the method was performed in human plasma and experiments performed in mouse plasma, composition of control mice, and human plasma samples was previously compared.

The assay was considered satisfactory if precision, expressed as relative standard deviation (RSD) or coefficient of variation (%CV), was less than 15% for within- and between-run variation. Quality control samples were independently prepared from the calibration standards, according to Section 2.4. The samples contained three different concentrations of moxifloxacin: near the lower limit of quantification (LOQ), mid-point of the concentration range and near the upper LOQ. Two replicates of each control sample were processed and analyzed over three validation days. The assay precision has been calculated using the RSD and a one-way analysis of variance. The RSD for the lower LOQ was set at <20%.

Linearity was assessed by plotting calibration curves in human plasma and duplicated in three separate runs. Calibration was done by spiking different moxifloxacin concentrations in plasma and CNS tissue with solutions: between 5 and 250 *μ*g/L (5, 10, 50, 75, 100, 150, 175, 200, and 250 *μ*g/L) and between 0.1 and 2.5 *μ*g/g of brain (0.1, 0.5, 0.75, 1.0, 1.5, 1.75, 2.0, and 2.5 *μ*g/g of brain). The curves were fitted by a linear regression method through measurement of the peak area ratio of moxifloxacin into the internal standard solution.

Recovery of moxifloxacin was determined by comparing the postextraction quality control samples mean peak area ratio to the mean of the methanol standards (*n* = 9). Recovery was determined in three different runs at three concentrations (quality controls).

Moxifloxacin stability was acquired by analyzing replicates (*n* = 3) at three different concentrations (quality control samples). The following stability conditions were evaluated for each method: long-term stability at −20°C, −80°C, three freeze-thaw cycles, and 18 h after sample treatment in the autosampler. An interval of ±15% on either side of the initial concentration was applied to assess analyte stability.

### 2.5. Pharmacokinetic Application

#### 2.5.1. Experimental Models

BALB/c female mice, 7-8 weeks old, purchased from Elevage Janvier (Le Genest-St- Isle, France) were used. The virulent strain *L. monocytogenes* EGDe [17] was provided by the bacteriology laboratory of the Pasteur Hospital (Paris, France) and used to induce listeriosis in these animals.

Mice were weighed (23 to 27 g), then injected intravenously via the lateral tail vein with 1 × 10^5^ *L. monocytogenes* in 0.5 mL of saline isotonic solution to induce listeriosis. At 36 hours after infection, mice were treated with an intraperitoneal (i.p.) injection of moxifloxacin (50 mg/kg, 0.1 mL). This protocol was approved by the Animal Welfare Committee of the Pasteur Institute.

#### 2.5.2. Sample Collection

The pharmacokinetic profiles of moxifloxacin in mouse plasma and brain tissue (infected or not) were analyzed immediately after a single dose of moxifloxacin. Moxifloxacin concentrations were measured from samples collected at 0, 5, 15, 30, 60, 120, 240, 360, and 480 minutes after a single-drug injection. At each sampling time, 6 animals per group were sacrificed.

Brains were washed in sterile saline to clear the circulating blood, then dried, weighed (0.5 g ± 0.01 g), crushed, and homogenized aseptically and centrifuged. Plasma was obtained from blood by centrifugation. Cerebral supernatants and plasma samples were frozen immediately and stored at −80°C until analysis.

#### 2.5.3. Pharmacokinetic Analysis

Concentration time data were analyzed using a noncompartmental model with zero-order absorption and first-order elimination via a nonlinear least squares technique. Pharmacokinetic parameters were estimated through standard methods. Maximal plasma and brain concentrations (*C*_max⁡_) were determined directly by inspection of the experimental concentration time curves, the values for the area under the concentration time curve (AUC_t_) were calculated by using the trapezoidal rule until the last concentration was measured. Penetration rate (AUC brain/AUC plasma) and the ratio sizing pharmacokinetic parameter variations between control and infected mice were also calculated. Results are expressed as means ± standard deviation.

## 3. Results

### 3.1. Validation of the Method

**Figure 2:**
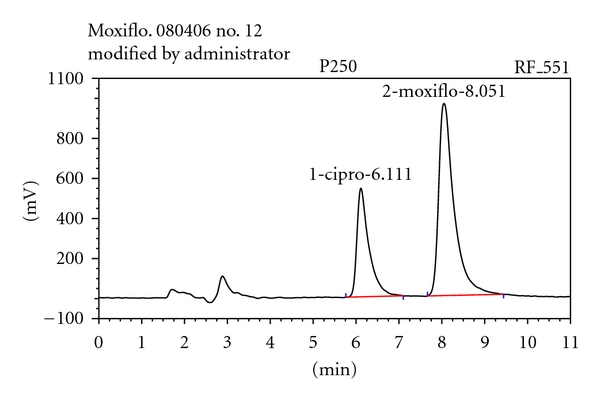
Chromatogram of moxifloxacin (retention time = 8.0 min) and the internal standard ciprofloxacin (retention time = 6.0 min) in plasma (concentration = 250 *μ*g/L).

Following the assay conditions described above, moxifloxacin was separated from the internal standard, ciprofloxacin; retention times, were 6.0 and 8.0 minutes, respectively (Figure 2). The chromatograms obtained from the analysis of two blank matrices show no interfering peaks with the same retention times and no interfering peak was observed for the retention times of the two fluoroquinolones from human plasma, mouse plasma, and mouse brain. The resolution between ciprofloxacin and moxifloxacin was up to 2.

The calibration curve obtained by plotting peak: area ratio (moxifloxacin/internal standard) versus concentration was linear over the range 5 to 250 *μ*g/L in plasma and 0.1 to 2.5 *μ*g/g in cerebral tissue. Linearity was observed in our moxifloxacin analyses from plasma and brain tissue (*r* ^2^ = 0.999 for both) over the evaluated concentration ranges. The plasma quantification lower limit was 5.0 *μ*g/L and 0.1 *μ*g/g for brain, matching the calibration curves first point. Excellent results were obtained for precision (CV < 10%; CV < 5%) and accuracy (99.2–111.5%; 94.8–106.2%) for plasma and brain tissue, respectively (*n* = 6 for each control; Tables 1(a) and 1(b)).

The mean moxifloxacin extraction recovery from plasma and cerebral tissue was above 75% (data not shown). The stability experiment was carried out under four conditions. Triplicates of each quality control sample were analyzed. Specimens stored at −20°C and at −80°C were stable for at least 4 months (data not shown). Moxifloxacin remained stable over three freeze-thaw cycles (between-run variation rate <11% for plasma and <10% for brain, and recovery ranged from 99% to 103% for plasma and from 100% to 105% for brain). Moxifloxacin also remained stable 18 h after sample treatment (CV < 5%, recovery between 98% to 105% for plasma and 99% to 106% for brain).

### 3.2. Pharmacokinetic Study

**Figure 3:**
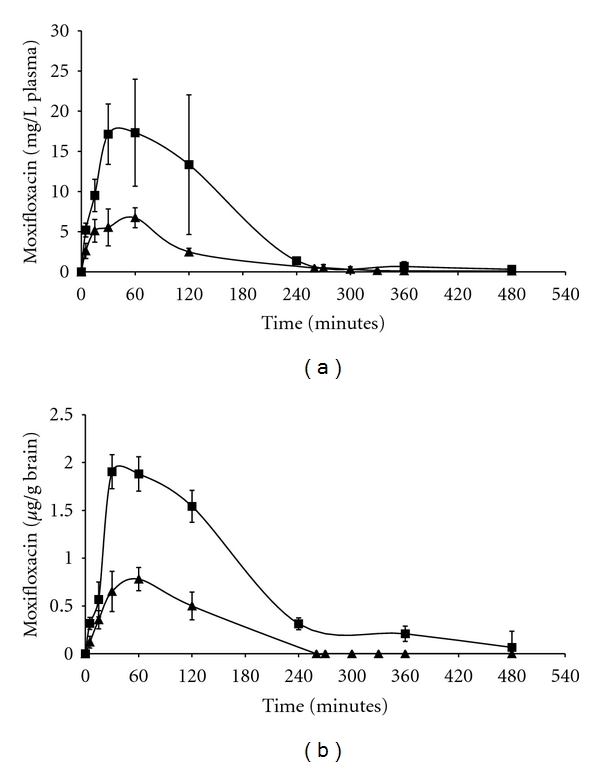
(a) Plasma concentration time course (mg/L) of moxifloxacin in mice (infected ■ or not ▲) after a single i.v. administration of 50 mg per kg of bodyweight (bolus injection) (*n* = 6); (b) brain concentration time course (*μ*g/g of brain) of moxifloxacin in mice (infected ■ or not ▲) after a single i.v. administration of 50 mg per kg of body weight (bolus injection) (*n* = 6).

The methods described above were utilised in the determination of mean moxifloxacin concentrations from plasma and the CNS after a single i.p. injection (50 mg/kg) in *L. monocytogenes* infected and control mice. The pharmacokinetic profiles are represented in Figures 3(a) and 3(b), and the parameters determined for moxifloxacin are shown in Table 2.

In plasma, for infected mice, the moxifloxacin *C*_max⁡_ was reached at 0.75 h (*C*_max⁡_ 17.3 ± 6.6 mg/L) versus 1 h (*C*_max⁡_ 6.7 ± 1.2 mg/L) for the control group. Nevertheless, after 1 hour, the concentration of moxifloxacin rapidly dropped and became undetectable after 8 hours (versus 6 hours for the control group). The plasma AUC_24_ was 27.1 mg·h/L and 9.5 mg·h/L in infected and control mice, respectively. The AUC_24_/MIC ratio (AUIC) for moxifloxacin was 54, and the *C*_max⁡_/MIC ratio was 34 in infected mice.

In cerebral tissue, moxifloxacin was quickly detected (5 minutes) and its concentration peaked at 2.0 ± 0.2 *μ*g/g (compared with 0.8 ± 0.3 *μ*g/g in the control group) within 1 hour of i.p. administration. After 0.75 h, the cerebral concentration of moxifloxacin decreased, but was still detectable 8 hours after administration (versus 6 hours in controls).

The cerebral AUC_24_ was 3.3 *μ*g·h/g and 1.0 *μ*g·h/g in infected and control mice, respectively. The AUC_brain_/AUC_plasma_ ratio was always greater than 0.1.

## 4. Discussion

In this study, we described a HPLC method to quantify moxifloxacin penetration in plasma and brain. Optimization of the method of Ba et al. was performed by modifying several preanalytical and analytical steps [9]. In particular, we assessed and fine-tuned the pH and composition of the mobile phase, SDS concentration, addition of TBABr, and detection mode. Mouse plasma and brain samples were processed by deproteinization with acetonitrile due to the low-protein binding of moxifloxacin [1]. Due to the extraction efficiency, we decided to validate the method using ciprofloxacin as an internal standard.

Separation was achieved by ion-pairing reversed-phase chromatography with a 10 mM concentration of SDS [11]. Liang et al. showed that above this concentration, fluoroquinolone resolution was not improved and column equilibration time was optimum. Competing-base agent (TBABr) was added at the concentration of 10 mM to increase resolution between moxifloxacin and ciprofloxacin, and to improve the peak shape [9, 11]. The pH of the mobile phase was fixed at 3.5 with citric acid buffer at a concentration of 25 mM [11]. Finally, the optimized mobile phase consisted of 10 mM TBABr, 10 mM SDS, 25 mM citric acid, 40% acetonitrile, and 3% methanol at pH 3.5. Due to the properties of moxifloxacin and ciprofloxacin, fluorescence detection was used because of its better sensitivity and specificity than UV detection in complex matrix for pharmacokinetic studies.

The HPLC method based on standard chromatographic conditions, rapid sample preparation and analysis (less than 10 min) has been developed for a dual-matrix moxifloxacin determination (plasma and cerebral tissue). The lower LOQ was found to be 5 *μ*g/L in plasma and 0.1 *μ*g/g in brain. The LOQ in plasma is similar to that reported in recent studies [10, 18]. Moreover, we did not need derivatization with a fluorescent molecule [19]. In plasma and brain tissue, it is low enough to enable pharmacokinetic studies in mice, other animals, or humans. This HPLC method is helpful and essential to study the pharmacokinetics and cerebral penetration of moxifloxacin. Several methods have been described for moxifloxacin quantification in different matrices (plasma, urine, cerebrospinal fluid, peritoneum, lung, etc.) [11, 14, 20]. However, most of these methods are described for only one matrix and some of them have very high LOQ (90 *μ*g/L for the method described by Hemanth Kumar and Ramachandran) [18] which are not suitable for pharmacokinetic studies in mice.

To our knowledge, this study is the first to evaluate moxifloxacin brain penetration by measuring concentrations in a murine model of cerebral listeriosis after i.p. treatment. Rodriguez-Cerrato et al. determined moxifloxacin CSF penetration in a rabbit model of *Escherichia coli* meningitis [14]. Kanellakopoulou et al. showed the pharmacokinetics of moxifloxacin in noninflamed CSF of humans [13, 14]. These two studies investigated moxifloxacin pharmacokinetics in CSF. In contrast, we have investigated moxifloxacin pharmacokinetics in plasma and brain tissue due to the intracellular development of *L. monocytogenes*. Subsequently, we have shown modification of moxifloxacin pharmacokinetics between infected or control mice (plasma and brain data).

Moxifloxacin brain penetration was shown according to its presence in CSF, as highlighted by Rise et al. [21]. Indeed, plasma and CNS tissue kinetics increased by 2.9 and 3.3 times, respectively, between control and infected mice (*C*_max⁡_, half-life, AUC_0–24 h_). Inflammation of the brain-blood barrier could explain the increase of these parameters in brain but not in plasma. The increase in plasma could be due to a diminution of hepatic metabolism or renal elimination due to infection by *L. monocytogenes*.

Our study is not without limitations including the omission of weighing animals before and after infection with *L. monocytogenes* to determine potential dehydration, which could account for the increase in plasma concentrations of moxifloxacin in infected compared with control mice. Also, we did not quantitate the concentration of moxifloxacin in the CSF of mice to distinguish between diffusion in the blood-brain barrier and diffusion in brain tissue. Finally, we did not determine the concentration of moxifloxacin in urine; a reduction in renal elimination of moxifloxacin in infected mice might explain the increased serum moxifloxacin concentration in infected animals.

As Grayo et al. showed, AUIC (AUC_plasma_/MIC) is the most relevant parameter to demonstrate *in vivo* efficiency. For fluoroquinolones, an AUIC value that appears sufficient *in vitro* and in animal and clinical trials, and for other infections, is between 30 [22] and 125 [23]; in our study it was equal to 54 and could explain the efficacy of moxifloxacin in CNS listeriosis [7]. In contrast to Alffenaar et al., we assessed cerebral penetration by determination of the AUC_brain_/AUC_plasma_ ratio, and not the AUC_CSF_/AUC_plasma_ ratio, which is the more relevant quantitative marker of antibacterial diffusion in tissue [24]. These ratios were always greater than 0.1, varying from plasma to brain. Even if the AUC_brain_/AUC_plasma_ ratio is not high, the AUIC in brain is enough to explain the *in vivo *efficacy of moxifloxacin [7]. Subsequently, we showed diffusion of moxifloxacin (5 minutes after injection) in cerebral tissue in infected mice unlike amoxicillin, considered in [6]. This rapid diffusion is probably related to increased blood-brain barrier permeability due to the inflammation of the meninges resulting in prolonged detection of the drug in the brain tissue [7, 21, 25]. Moreover, moxifloxacin can be detected 8 hours after administration in infected mice and 6 hours in control mice. This confirms the good level of brain penetration of moxifloxacin as demonstrated by Wise et al. [21] and it could explain the *in vivo* efficiency [7].

## 5. Conclusion

We have developed and validated a HPLC moxifloxacin determination method in plasma and brain tissue in a mouse model. Based on this sensitive method, we demonstrated moxifloxacin penetration in the CNS for the first time in this listeriosis model. As expected, we suggest that infection can modulate the pharmacokinetic profile of moxifloxacin. Indeed, the higher antimicrobial concentrations can be correlated with improved diffusion into infected tissue (plasma and cerebral) in listeriosis than in a healthy model. This could explain the systemic antimicrobial *in vivo* efficiency of moxifloxacin against *L. monocytogenes*. Further, clinical data are required to confirm these findings.

Due to an excellent separation efficiency, sensitivity by fluorescent detection and its simplicity, this HPLC method could potentially meet the analysis requirements for any other complex tissues (such as liver or lung), or for therapeutic drug monitoring of moxifloxacin in other human diseases (such as cholecystitis or tuberculosis).

## Figures and Tables

**Table:** (a)

Nominal concentration (*μ*g/L)	Measured concentration (*μ*g/L)	Coefficient of variation (%)	Accuracy (%)	Measured concentration (*μ*g/L)	Coefficient of variation (%)	Accuracy (%)
		Within-run			Between-run	
20	22.3 ± 0.3	1.5	111.5	19.9 ± 1.9	9.5	99.0
125	125.5 ± 1.3	1.0	100.4	125.6 ± 2.8	2.2	100.5
225	240.4 ± 3.6	1.5	106.8	230.9 ± 8.1	3.5	102.6

**Table:** (b)

Nominal concentration (*μ*g/g)	Measured concentration (*μ*g/g)	Coefficient of variation (%)	Accuracy (%)	Measured concentration (*μ*g/g)	Coefficient of variation (%)	Accuracy (%)
		Within-run			Between-run	

0.2	0.19 ± 0.01	3.6	94.8	0.21 ± 0.01	5.0	105.5
1.25	1.33 ± 0.03	2.5	106.2	1.30 ± 0.04	3.0	104.3
2.25	2.17 ± 0.03	1.4	96.6	2.26 ± 0.11	5.0	100.4

**Table 2:** Plasma and cerebral pharmacokinetic parameters after intraperitoneal administration of moxifloxacin (50 mg/kg) in infected and control mice (*n* = 6).

	*T*_max⁡_ (h)	*C*_max⁡_	*T*_1/2_ (h)	Cl (L/h)	Vd (L/kg)	AUC_0-24h_
Plasma		(mg/L)				(mg·h/L)
Infected	0.75	17.3 ± 6.6	1.9	0.02	3.5	27.1
Control	1	6.7 ± 1.2	1.1	0.08	6.5	9.5

Brain tissue		(*μ*g/g of brain)				(*μ*g·h/g of brain)
Infected	0.75	2.0 ± 0.2	21.7			3.3
Control	1	0.8 ± 0.3	10.7			1.0
